# ELM 2016—data update and new functionality of the eukaryotic linear motif resource

**DOI:** 10.1093/nar/gkv1291

**Published:** 2015-11-28

**Authors:** Holger Dinkel, Kim Van Roey, Sushama Michael, Manjeet Kumar, Bora Uyar, Brigitte Altenberg, Vladislava Milchevskaya, Melanie Schneider, Helen Kühn, Annika Behrendt, Sophie Luise Dahl, Victoria Damerell, Sandra Diebel, Sara Kalman, Steffen Klein, Arne C. Knudsen, Christina Mäder, Sabina Merrill, Angelina Staudt, Vera Thiel, Lukas Welti, Norman E. Davey, Francesca Diella, Toby J. Gibson

**Affiliations:** 1 Structural and Computational Biology, European Molecular Biology Laboratory, Meyerhofstrasse 1, 69117 Heidelberg, Germany; 2 Health Services Research Unit, Operational Direction Public Health and Surveillance, Scientific Institute of Public Health (WIV-ISP), 1050 Brussels, Belgium; 3 Ruprecht-Karls-Universität, Heidelberg, Germany; 4 Conway Institute of Biomolecular and Biomedical Sciences, University College Dublin, Dublin 4, Ireland

## Abstract

The Eukaryotic Linear Motif (ELM) resource (http://elm.eu.org) is a manually curated database of short linear motifs (SLiMs). In this update, we present the latest additions to this resource, along with more improvements to the web interface. ELM 2016 contains more than 240 different motif classes with over 2700 experimentally validated instances, manually curated from more than 2400 scientific publications. In addition, more data have been made available as individually searchable pages and are downloadable in various formats.

## INTRODUCTION

**Figure 1.**
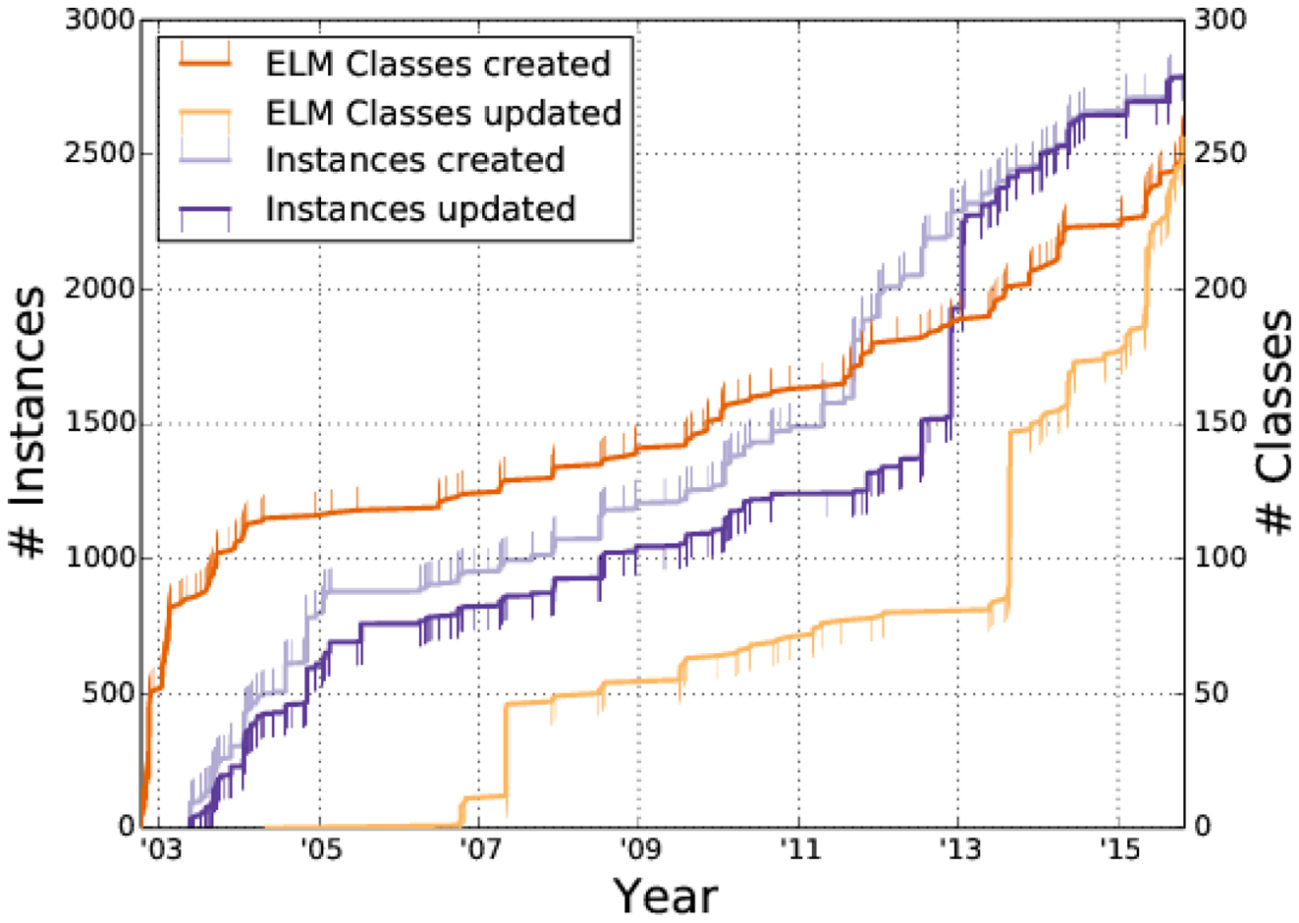
ELM history showing the creation date (red line) and date of latest modification (orange line) of each ELM motif class (light blue line) and motif instance (dark blue line) annotated in the ELM database. Note that for each entry only the very latest modification time is recorded, not every modification timestamp.

Short Linear motifs (SLiMs) are small protein-interaction-mediating modules that have unique properties (1,2). They play an important role in biological systems (3–6) and the analysis of motifs in protein sequences remains an important step in protein research. The ELM database provides manually curated classes of motifs as well as instances thereof. Furthermore, it provides a web interface that allows users to explore possible instances of annotated classes in proteins of interest. More than 10 years since its inception (7,8) it remains a popular resource being continually used by scientists worldwide. It has proved invaluable for investigating interactions mediated by short linear motifs, be it for individual proteins (9–11), large-scale analyses (12–14), algorithm development (15), prediction of novel motif-mediated interactions (16) or investigation of host–virus interactions (17–20). Here we give an overview of the latest developments and new features introduced to the ELM resource (see Figure 1) since the last update (21).

## RESOURCE DESCRIPTION

The ELM resource provides two main services, a database of short linear motif annotation and a tool that uses this information to explore possible instances of motifs in any given protein sequence. The main database content are annotations of motif classes, hand-curated from the scientific literature and enriched with links to resources such as PSI-MI (22), UniProt (23), GO (24), PDB (25), switches.ELM (6), Interpro (26), iELM (27), Pfam (28) and KEGG (29).

An ELM motif class is described by a unique regular expression pattern, such as ‘...([ST])P.’ (meaning any three residues followed by a serine or threonine, followed by proline and another wildcard residue) for the DOC_WW_Pin1_4 class. Ideally, each motif class has multiple example instances of this motif annotated, whereby an instance is described as a match to the regular expression pattern of the ELM motif class in a protein sequence. For each instance entry, ideally, multiple sources of experimental evidence are recorded (identifying participant, detecting motif presence and detecting interaction), and, following annotation best practices, a reliability score is given by the annotator.

## NEW FEATURES AND FUNCTIONALITY

### Interface

**Figure 2.**
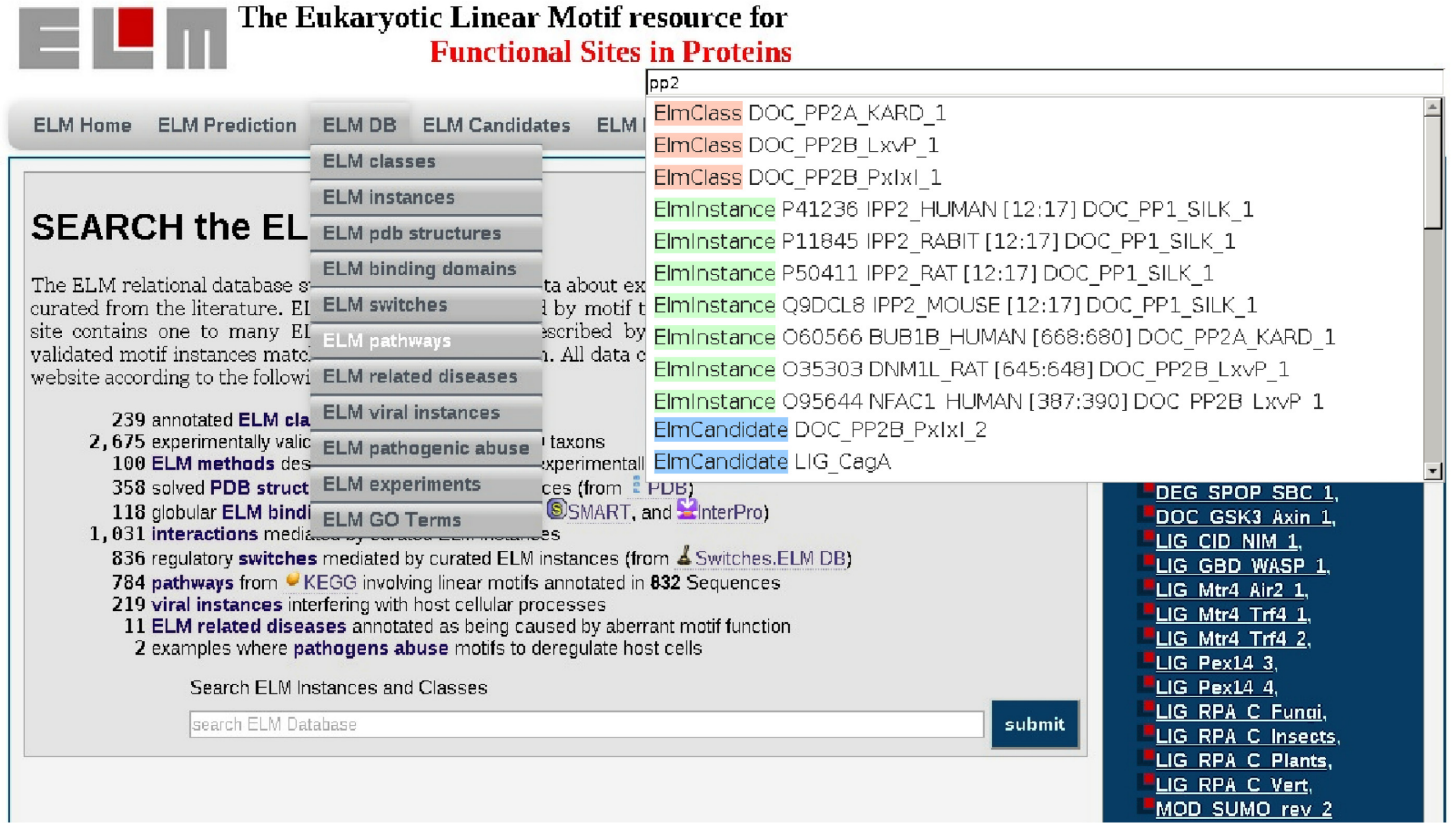
Screenshot of the ELM home page showing autocompletion of keyword search and expanded menu bar. The dropdown menu lists individual pages of ELM database content; the top right shows a dropdown of the autocompletion search for the keyword ‘pp2’, different content type is highlighted in different colors (ELM classes: orange, ELM instances: green, ELM class candidates: blue); a click on a link directs to the details page of the entry.

The HTML interface has been updated and new functionality has been added. A multi-tier navigation menu has been implemented to provide improved accessibility to the individual pages. Also, on each page, a unified search box is available, providing auto-completion and faster access to the ELM database content via simple keyword search (see Figure 2).

Several new webpages have been introduced to allow easier access to the database content. There are now individual pages for interaction domains, motif-mediated switches, PDB structures, ELM methods and GO terms. This allows easy access to individual content searching and selecting data by user provided keywords, fostering data dissemination and re-use.

**Table 1.** Summary of data stored in the ELM database^1^

Functional sites		ELM classes		ELM instances		PDB structures		GO terms		PubMed links	
Total	159		246		2702		348		549		2439
By category		LIG	137	Human	1594						
		MOD	31	Mouse	253			Biological Process	283	From class	1174
		DEG	25	Rat	130						
		DOC	22	Yeast	94			Cellular Compartment	119	From instance	1746
		TRG	20	Fly	90						
		CLV	11	Other	541			Molecular Function	147		

^1^ as of November 2015.

**Table 2.** 49 novel^1^ ELM classes that have been added since the last ELM publication (21), together with the number of associated instances (middle column) and a short description

ELM class identifier	Instances	ELM class description
DEG_Kelch_KLHL3_1	4	An acidic degron motif present in wnk kinases necessary to interact with kelch domain of KLHL2 and KLHL3 proteins for efficient ubiquitination degradation.
DEG_Kelch_Keap1_1	13	Motif that binds to the Kelch domain of KEAP1 with high affinity. This high affinity motif is required for the
DEG_Kelch_Keap1_2	1	efficient recruitment of target proteins to the Cul3-based E3 ligase.
DEG_Kelch_actinfilin_1	1	A hydrophobic degron motif present in some kainate receptors necessary to interact with kelch domain of actinfilin protein for efficient ubiquitination and degradation.
DEG_Nend_Nbox_1	0	N-terminal motif that initiates protein degradation by binding to the N-box of N-recognins. This N-degron variant comprises a bulky hydrophobic residue as destabilizing residue.
DEG_Nend_UBRbox_1	0	N-terminal motifs that initiate protein degradation by binding to the UBR-box of N-recognins. Four different
DEG_Nend_UBRbox_2	0	N-degron variants comprise different N-terminal residues. Type I destabilizing residues can either occur as primary
DEG_Nend_UBRbox_3	0	destabilizing residues, which are positively charged amino acids directly recognized by N-recognins, or as
DEG_Nend_UBRbox_4	8	secondary and tertiary destabilizing amino acids, which can be conjugated to a primary destabilizing residue.
DEG_SPOP_SBC_1	8	The S/T rich motif known as the SPOP-binding consensus (SBC) of the MATH-BTB protein, SPOP, is present in substrates that undergo SPOP/Cul3-dependant ubiquitination.
DOC_CKS1_1	8	Phospho-dependent motif that mediates docking of CDK substrates and regulators to cyclin-CDK-bound Cks1.
DOC_GSK3_Axin_1	6	Docking motif present in Axin protein binds the GSK-3β kinase and aids the phosphorylation of components in the APC destruction complex.
DOC_PP1_MyPhoNE_1	9	Docking motif that binds to the catalytic subunit of Protein Phosphatase 1 (PP1c).
DOC_PP1_SILK_1	14	Protein phosphatase 1 catalytic subunit (PP1c) interacting motif that often cooperates with and is located N-terminal to the RVXF motif to dock proteins to PP1c.
DOC_PP2A_KARD_1	1	Protein Phosphatase 2A (PP2A)-binding motif found in BubR1 for docking to the regulatory subunit B56 of PP2A.
DOC_PP2B_LxvP_1	8	Docking motif in calcineurin substrates that binds at the interface of the catalytic CNA and regulatory CNB subunits.
DOC_USP7_UBL2_3	0	The USP7 CTD domain binding motif variant based on the ICP0 and DNMT1 interactions.
LIG_Axin_LRP6_1	0	Motif in LRP6, which in its phosphorylated form binds Axin in a pseudo-substrate manner.
LIG_CID_NIM_1	1	The NIM motif in Trf4 interacts with the CTD-interacting domain (CID) of Nrd1.
LIG_CNOT1_NIM_1	10	The CNOT1-interacting motif (NIM) found in Nanos proteins mediates recruitment of the CCR4-NOT deadenylase complex.
LIG_CaMK_CASK_1	6	Motif mediating binding to the calmodulin-dependent protein kinase (CaMK) domain of the membrane protein CASK/Lin2.
LIG_DCNL_PONY_1	2	DCNL PONY domain binding motif variant based on the UBE2M and UBE2F interactions.
LIG_EF_ALG2_ABM_1	9	This isoform-specific ALG-2-binding motif binds to the EF hand domains of the proapoptotic Ca^2 +^-binding
LIG_EF_ALG2_ABM_2	3	ALG-2 protein in a calcium-dependent manner.
LIG_FZD_DVL_PDZ	0	A short internal motif near the C-terminus of Frizzleds, which interacts with the PDZ domain of DVL in Wnt pathway.
LIG_GBD_WASP_1	4	A hydrophobic motif of double function – it acts as an autoinhibitory element of the GTPase- binding domain (GDB), as well as mediating the protein's interactions with the Arp2/3 complex.
LIG_GSK3_LRP6_1	8	Motif present five times on membrane receptor LRP6, responsible for GSK3 binding and inhibition when phosphorylated.
LIG_LIR_Apic_2	1	Apicomplexa-specific variant of the canonical LIR motif that binds to Atg8 protein family members.
LIG_LIR_Gen_1	21	Canonical LIR motif that binds to Atg8 protein family members to mediate processes involved in autophagy.
LIG_LIR_LC3C_4	1	Non-canonical variant of the LIR motif that binds to Atg8 protein family members to mediate processes involved in autophagy.
LIG_LIR_Nem_3	1	Nematode-specific variant of the canonical LIR motif that binds to Atg8 protein family members.
LIG_LRP6_Inhibitor_1	0	Short motif present in extracellular of some Wnt antagonists recognized by the N-terminal β-propeller domain of LRP5/6 and thus inhibits the Wnt pathway.
LIG_Mtr4_Air2_1	3	This motif on Air2 interacts with the DExH core of Mtr4, forming a part of the nucleus-located TRAMP complex.
LIG_Mtr4_Trf4_1	4	This motif on Trf4 interacts with the DExH core of Mtr4, forming a part of the nucleus-located TRAMP complex.
LIG_Mtr4_Trf4_2	3	This motif on PAPD5 interacts with the DExH core of SKIV2L2, forming a part of the nucleus-located TRAMP complex.
LIG_Pex14_3	1	Motif in Pex5 interacting with the N-terminal domain (NTD) of Pex14
LIG_Pex14_4	0	Fungal motif in Pex5 interacting with the N-terminal domain of Pex14
LIG_RPA_C_Fungi	1	Fungi version of the RPA interacting motif.
LIG_RPA_C_Insects	0	Insect version of the RPA interacting motif.
LIG_RPA_C_Plants	0	Plant version of the RPA interacting motif, which is located on DNA replication and repair proteins UNG2, XPA, TIPIN, SMARCAL1 and RAD14 and interacts with Replication Protein A (RPA), a DNA binding protein.
LIG_RPA_C_Vert	4	The RPA interacting motif is located on DNA replication and repair proteins UNG2, XPA, TIPIN, SMARCAL1 and RAD14 and interacts with Replication Protein A (RPA), a DNA binding protein.
LIG_SUFU_1	5	A hydrophobic motif in GLI transcription factors required for binding to SUFU protein, which inhibits their activity and hence negatively regulates hedgehog signalling.
LIG_UBA3_1	2	UBA3 adenylation domain binding motif variant based on the UBE2M and UBE2F interactions.
LIG_WD40_WDR5_VDV_1	3	This WDR5-binding motif binds to a cleft between blades 5 and 6 of the WD40 repeat domain of WDR5, opposite
LIG_WD40_WDR5_VDV_2	2	of the Win motif-binding site, to mediate assembly of histone modification complexes.
LIG_WD40_WDR5_WIN_1	7	Known as the Win (WDR5 interaction) motif, this peptide contains an invariant arginine residue that inserts into the
LIG_WD40_WDR5_WIN_2	4	central tunnel of the WD40 repeat domain of WDR5 to mediate assembly of histone modification complexes.
LIG_WD40_WDR5_WIN_3	3	Surrounding this arginine are small residues that fit tightly at the entrance of the arginine-binding pocket.
MOD_SUMO_rev_2	20	Inverted version of SUMOylation motif recognized for modification by SUMO-1

^1^ as of November 2015.

### Database content

**Figure 3.**
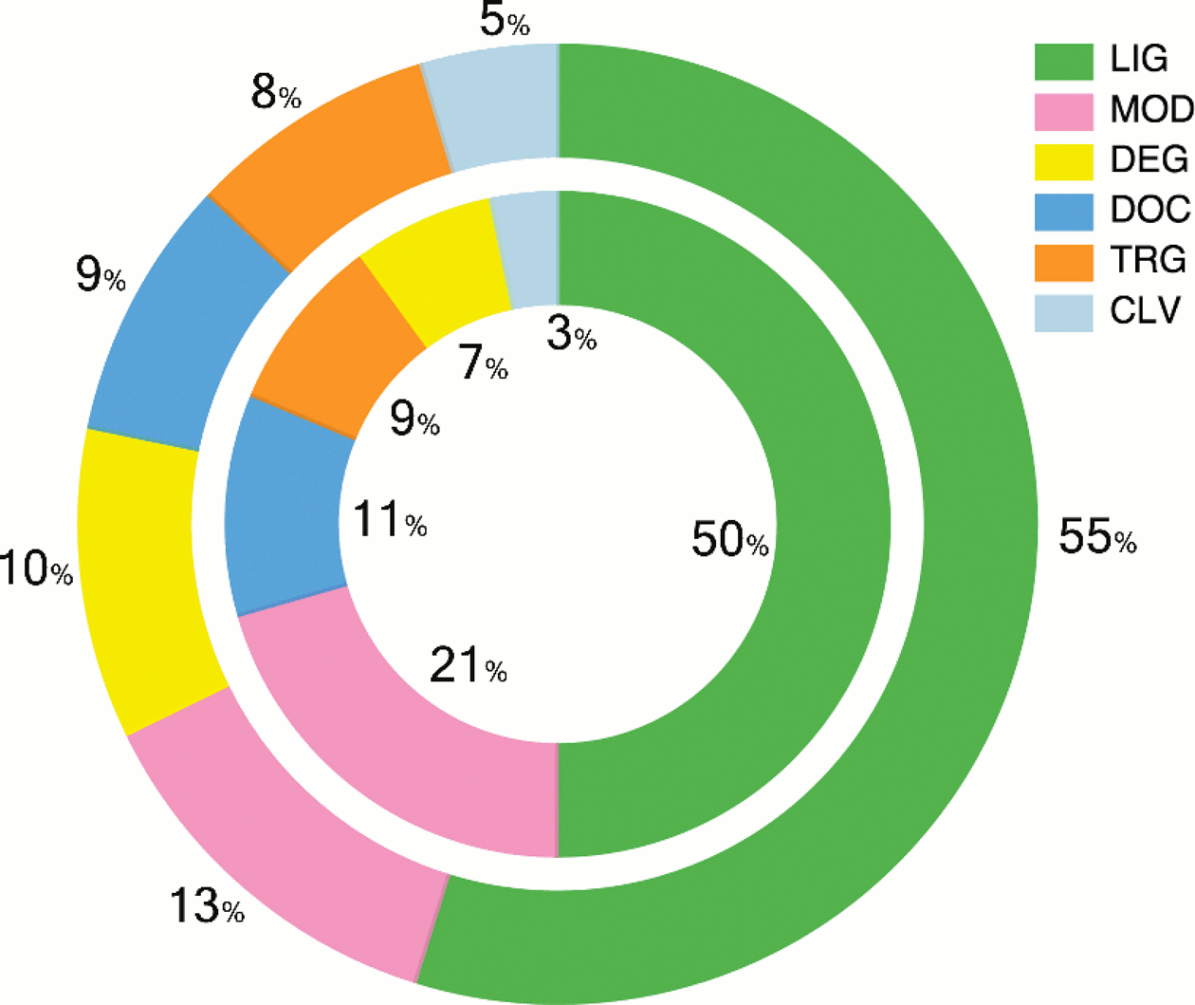
Percentages of ELM classes (outer ring) and instances (inner ring) by type.

**Figure 4.**
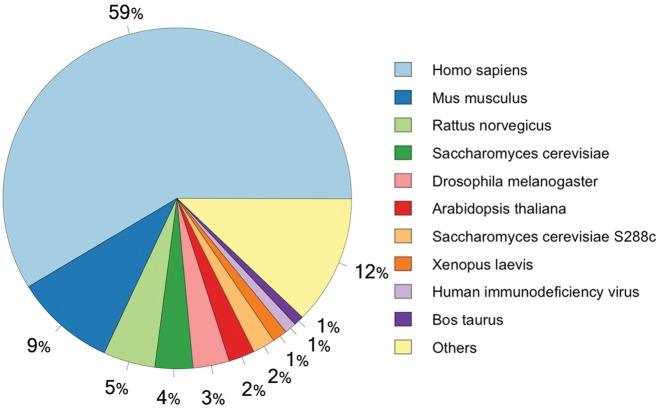
Taxons annotated in the ELM database ordered by number of instances (in percentages).

**Figure 5.**
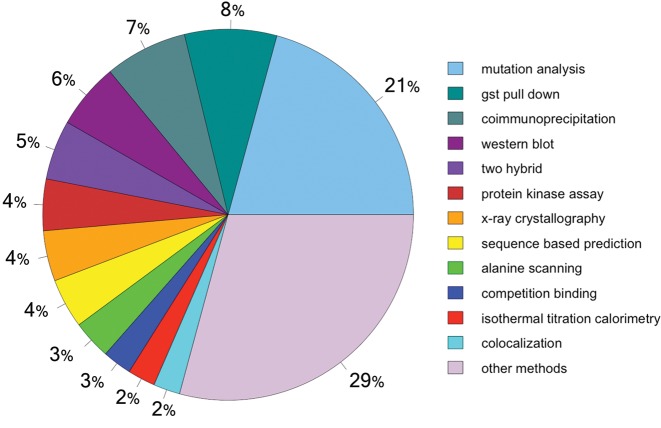
Methods used for ELM annotation, ordered by number of motif instances for which the method has been annotated (in percentages). A full list can be found at http://elm.eu.org/infos/browse_elm_methods.html, which includes links to instances and PSI-MI vocabulary (22).

The ELM database has been updated and existing data types have been enriched with more data, see Tables 1 and 2, Figures 1 and 3 for an overview of data on ELM classes and instances, as well as Figures 4 and 5 for information about the taxonomic distribution of ELM instances and the experimental methods annotated for these instances. Furthermore, new data types have been added: Many motif instances have been annotated from large-scale studies (30) for which known mutations in the motif contribute to diseases. These can be collectively viewed at the ELM disease page (http://elm.eu.org/infos/diseases.html). Each annotated disease is described by a short abstract, links to the sequence variation causing the condition (linked to swissprot (31)) in the the motif-bearing protein as well as reference article(s). Each entry is linked to the corresponding ELM instances page.

### Database and web server optimization

The ELM resource is implemented using a PostgreSQL (https://postgresql.org) relational database as a backend to store all annotations and associated data, while the frontend web interface makes use of the Django web-framework (https://djangoproject.com). Recent improvements include an updated annotation system allowing easy annotation and correction of entries. This aids annotators in inserting novel entries and updating existing ones. Figure 1 illustrates how this annotation system has helped increasing the database content: since its implementation (in the year 2014) many new ELM classes and instances have been annotated, and a large number of classes and instances have been revised.

The high degree of data integration and interconnectedness caused some ELM database queries to become slow. To remedy this and thus increase user experience, an HTTP Cache/Reverse Proxy has been employed caching rendered HTML pages, which significantly speeds up page delivery and increases user experience.

### Downloads

The data annotated in the ELM database is freely available to the scientific community and the ELM team tries to make this data as easily accessible as possible. Several new pages are now available providing more download formats/options; the best starting point for looking for ELM downloads is the web page http://elm.eu.org/downloads.html. Novel pages include the experimental methods used during annotation, the PDB structures associated with motif–domain interactions, or all linked GO-terms. All of these are also available for download in computer-parseable tab-separated format. Also, a simple timestamp has been implemented, which allows clients to update their data only when newer data is available. Further formats and options can be implemented upon request; the authors welcome suggestions to implement user-suggested features.

## SHORT LINEAR MOTIFS IN BIOLOGICAL PATHWAYS

**Figure 6.**
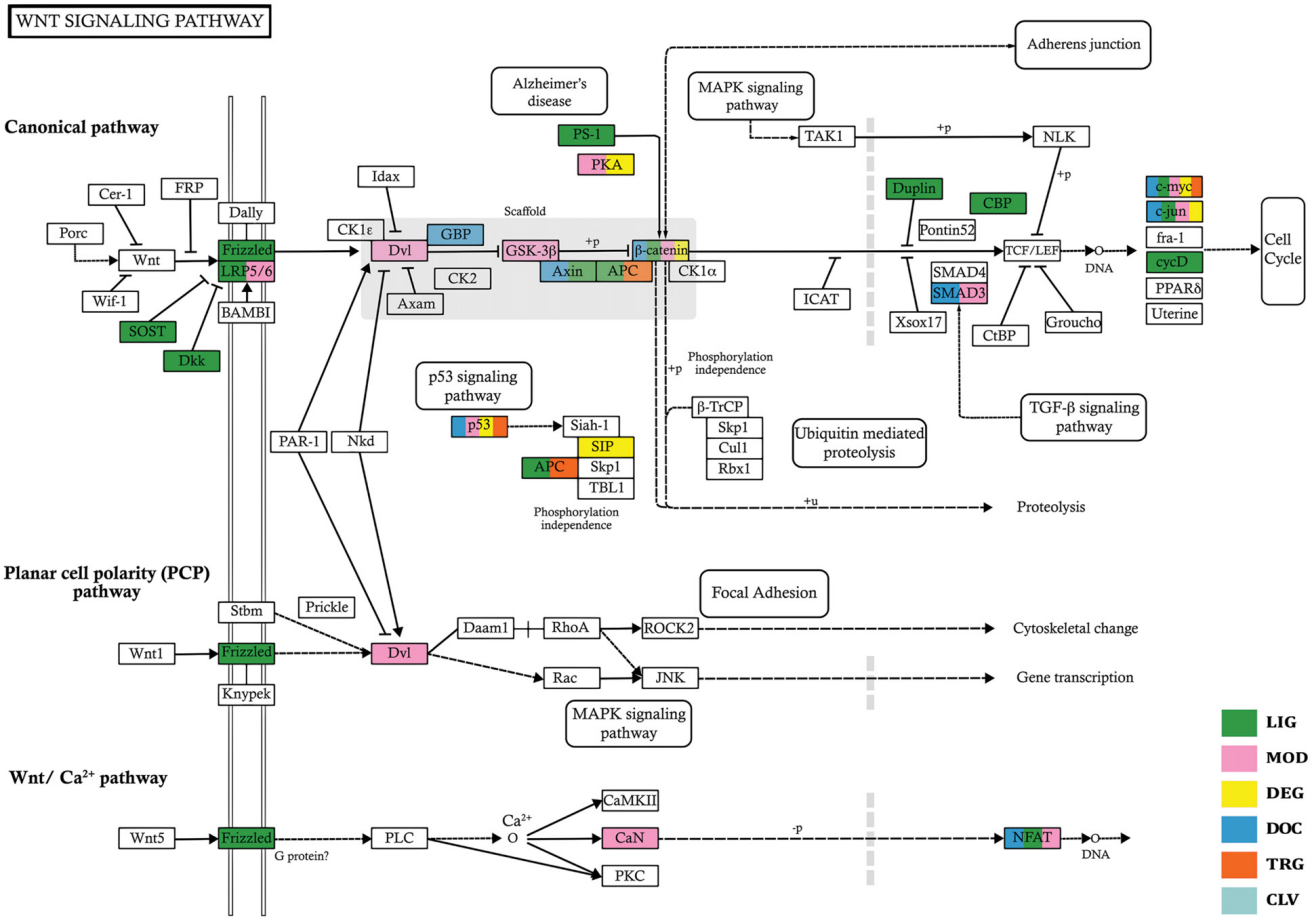
‘Wnt Signaling pathway’ from KEGG (29) (ID hsa04310). The color scheme has been modified for the cases having more than one ELM class in order to provide additional clarity. The original figure can be reproduced on the ELM web server (http://elm.eu.org/pathways/index.html?q=wnt)

Relevance of linear motifs is well known in signaling pathways (32). Furthermore, accumulation of more experimental evidence demands a systematic analysis of all biological pathways for the presence of linear motifs. The ELM resource assists in this endeavor by providing a distinct color mapping of different ELM classes on the proteins involved in different pathways, information of which is obtained from KEGG (29). We have used the Wnt signaling pathway (hsa04310) to illustrate this feature (Figure 6).

Signaling in this pathway starts from Wnt proteins that transduce the signal from the extracellular part of the cell to the interior via the Frizzled receptor, which in turn transmits it to other regulatory proteins. A key component of Wnt signaling is β-catenin which needs to accumulate in the cytoplasm in order to be translocated into the nucleus, where it subsequently induces a cellular response via gene transduction (33). This accumulation however is regulated by APC/Axin1 destruction complex in which β-catenin is ubiquitinylated (34). Thereafter, modified β-catenin is degraded via proteosomal machinery. Short linear motifs play a prominent role in this process as exemplified by the presence of at least four different motifs in β-catenin (annotated at the ELM resource): It contains modification sites for Casein kinase 1 (CK1) and glycogen synthase kinase 3 (GSK3) and the sequential modification of these sites by CK1 and GSK3 generates a phosphodegron at the N-terminus of this protein. This phospho-regulated degron acts as a recognition site for β-TrCP, a subunit of the SCF-β-TrCP E3 ligase enzyme (35). After binding to the activated degron, β-TrCP ubiquitinylates β-catenin, which gets subsequently degraded by the proteosomal machinery (36). Among other classes, β-catenin contains ligand binding (LIG) motifs for 14-3-3 proteins and the binding of 14-3-3 along with Chibby protein has been suggested to facilitate the nuclear export of β-catenin, which puts an end to its signaling (37). The presence of each of these motifs is necessary for modulation of this pathway and the regulation of β-catenin by different motif classes clearly underlines the important role of short linear motifs in signaling pathways.

## CONCLUSION AND FUTURE DIRECTIONS

The number of motifs that have been experimentally validated up to today is still small, with annotated instance numbers in the low thousands, compared to the estimated total number, which might exceed a million (38); hence, we expect many more motifs and motif-mediated interactions to be discovered (for guidelines on motif discovery see (39)). The ELM resource will continue to provide support to the scientific community with a repository of high quality annotations and facilitate the linear motif analysis of protein sequences.
